# Seats for Shop Girls

**Published:** 1881-08

**Authors:** 


SEATS FOR SHOP GIRLS.—We are glad to learn that a law has been enacted, requiring merchants and shop keepers to prepare seats for females in their business places.

Our readers, who began with the early issues of the journal, know that we have favored such an arrangement for female employees for some time past. Even young and strong men find that remaining on their feet from twelve to sixteen hours per day, as some of them are compelled to do, soon undermines the health of the most robust; and young women lose their strength and health much earlier than they. The reasons for this are so obvious as scarcely to require mention of them, further than the fact that females should be provided with seats, which they should be permitted to use from time to time, for at least five days of every month; and if their general health is to be preserved, as often at other times as fatigue from exertion demands that they should do so. A certain amount of rest is as essentially necessary to health as exercise, if the tone and integrity of muscular fiber is to be preserved and rendered useful in the affairs of life. The enlightened intelligence of the age is rapidly pointing out, or indicating many new, or heretofore unutilised methods and ways of preserving health, and thereby prolonging the period of human life, that should be heeded by the persons whom they specially interest or concern; and all reasonable assistance should be extended in carrying them into effect, by those having it in their power to do so.

We do not doubt but that the merchants who may prepare seats for their shop-women and girls will be gainers by it in the long run, as much, or more, than they would lose in their construction in the continued health and strength they would impart, and preserve to their employees. Nature is ever striving at equilibrium and compensation, and rest to the weary muscles and limbs of the often hardly-worked shop-girl is in harmony with her laws.

